# Robotics Education in STEM Units: Breaking Down Barriers in Rural Multigrade Schools

**DOI:** 10.3390/s23010387

**Published:** 2022-12-30

**Authors:** Angela Castro, Jhonny Medina, Cristhian A. Aguilera, Mario Ramirez, Cristhian Aguilera

**Affiliations:** 1 Instituto Especialidades Pedagógicas, Universidad Austral de Chile, Puerto Montt 5480000, Chile; 2 Departamento de Ciencias de la Ingeniería, Universidad de Los Lagos, Puerto Montt 5480000, Chile; 3 Instituto Politécnico Nacional, Ciudad de México 07738, Mexico; 4 Departamento de Ingeniería Eléctrica y Electrónica, Universidad del Bío-Bío, Concepción 4030000, Chile

**Keywords:** robotics, STEM education, multigrade education, digital divide

## Abstract

We report a novel proposal for reducing the digital divide in rural multigrade schools, incorporating knowledge of robotics with a STEM approach to simultaneously promote curricular learning in mathematics and science in several school grades. We used an exploratory qualitative methodology to implement the proposal with 12 multigrade rural students. We explored the contribution of the approaches to the promotion of curricular learning in mathematics and science and the perceptions of using robotics to learn mathematics and science. As data collection techniques, we conducted focus groups and semi-structured interviews with the participants and analyzed their responses thematically. We concluded that the proposal could contribute to meeting the challenges of multigrade teaching. Our findings suggest that the proposal would simultaneously promote the development of curricular learning in mathematics and science in several school grades, offering an alternative for addressing various topics with different degrees of depth.

## 1. Introduction

The rapid advance of the so-called STEM areas (science, technology, engineering, and mathematics) demands a prepared citizenry with new knowledge and skills. Children and young people’s education must consider the need to understand and participate in relevant problems of an interdisciplinary nature. Additionally, students must develop knowledge and skills that will allow them to grow in a society affected by the rapid development of technology in multiple areas [1]. However, the implementation of STEM education is a difficult task. It brings significant challenges, for example, agreement on what an integrated curriculum means and how to preserve the epistemic integrity of the areas to be integrated [2,3,4]. The difficulties associated with developing proposals that encourage this type of experience are especially challenging in rural educational contexts, especially if extreme cases of rural environments are considered, such as multigrade schools [5,6].

Multigrade schools are an extreme case of rurality in which students of multiple ages and grades receive their education in the same classroom [7,8]. In this type of school, students live in isolated geographical contexts or in small populations. The number of students in each school depends on the location. Thus, it is possible to find schools with three students at different levels that live far from the nearest city. Typically, a single teacher is in charge of the entire school, conducting the teaching processes for all students. The nature of multigrade teaching brings significant challenges under the current requirements of 21st-century citizenship that still need to be fully addressed [1,8,9].

This scenario implies a need to reduce the inequalities experienced by multigrade schools, both in access and knowledge and in the use of the latest technologies in classrooms. Technological tools are essential for equal rights and the achievement of digital literacy, which is considered a necessary skill for 21st-century citizens [10]. It is necessary to avoid a territorial digital divide that denies educational opportunities to rural students and limits their development possibilities [9].

Many countries have implemented educational policies to incorporate digital technologies into rural education [11,12] to fulfil constitutional rights and international obligations to ensure that all students have equal access to learning and development opportunities [7,12]. Despite these efforts, students in multigrade classrooms in these areas often struggle to meet learning standards in subjects such as language, mathematics, and science, as well as in developing digital skills [11]. As a result, it is necessary to explore new strategies to address these inequalities and improve student learning outcomes in multigrade classrooms in rural areas.

A viable way to incorporate the knowledge and use of technology in the classroom is through educational robotics [5,13]. Robotics is interdisciplinary and promotes the development of many 21st-century skills. Robotics has stimulated the development of various proposals seeking to incorporate it as a regular part of mathematics and science curricula so that all students can benefit from them [14,15,16,17]. These proposals have included guidelines linking robotics with curricula of a certain level in primary and secondary education. However, the proposals are not applicable when it is necessary to simultaneously incorporate robotics into other curricula, as in multigrade schools. Current proposals are intended for something other than this purpose and do not allow specific learning to be achieved at different school levels simultaneously during the same activity.

In this paper, we propose that one way to contribute to reducing the digital divide in multigrade schools is to implement integrated curricular units based on knowledge of robotics using a STEM approach to promote curricular learning in mathematics and science. For this purpose, we propose the incorporation of the knowledge and use of robotics to promote STEM learning in an integrated manner for multigrade schools. This proposal provides space for developing knowledge about robotics, such as sensors, actuators, and programming, and specific curricular learning in primary mathematics and science. Our objective is to explore the contribution of this proposal to promoting curricular learning in multigrade contexts and perceptions about the use of robotics as a means of learning mathematics and sciences in these contexts.

The main contributions of this work are the following:

- A method for incorporating robotics into the multigrade curriculum to promote STEM learning, which addresses the educational challenges of 21st-century citizens and helps reduce the digital divide, is proposed.
- We provide empirical evidence on the effectiveness of using educational robotics to enhance mathematics and science learning in multigrade schools.

The remainder of the paper is organized as follows: Section 2 describes related work concerning the challenges of rural multigrade schools and robotics in STEM education. Section 3 presents the course design and methodology. Section 4 describes the results regarding the promotion of STEM learning and the perceptions of using robotics. Section 5 discusses the principal findings and limitations of the study. Finally, Section 6 concludes by summarizing the primary results and providing suggestions for future research.

## 2. Literature Review

### 2.1. The Challenges of the Rural Multigrade School in the 21st Century

The rapid advances that have occurred in STEM knowledge have changed the knowledge and skills that citizens of the 21st century must possess [1]. Many of the challenges we face today as a society are interdisciplinary in nature and require the application of knowledge and skills associated with STEM. These new requirements, added to the imperatives of economic growth and global competitiveness, have put STEM education on the agenda of government initiatives in many countries [18]. STEM education is conceived as an approach that removes the barriers behind which science, technology, engineering, and mathematics are traditionally taught, integrating them into the real world through authentic and relevant experiences that combine them [19].

This scenario also affects the rural multigrade school and its community and poses the challenge of offering educational experiences that respond to these new demands of the 21st century to offer opportunities that impact the development of these communities [19]. The rural multigrade school is a pillar of its community. In disadvantaged rural sectors, it is a fundamental element in the construction and generation of cooperative and associative forms of social capital, which are transformed into resources and benefits for the inhabitants [20]. An interest in science, technology, and mathematics is engendered at an early age, and it is essential that all children, regardless of their educational context, participate in early experiences that awaken their interest in and knowledge about these areas [5], avoiding a territorial gap that denies development opportunities to rural students and limits their development opportunities [9].

However, teaching in rural multigrade contexts is not an easy task and brings with it significant challenges. Rural education is concentrated in primary schools, where, generally, only one teacher educates children from the first to the sixth grade at the same time [7,21]. The heterogeneity of these classrooms poses a great challenge for teachers, who must adapt the curriculum to several grades simultaneously in subjects that do not always progress similarly at the different school levels. As a consequence, many teachers teach up to six different classes at once, working on different subjects with each grade in turn, while the other grades work independently [6].

The complexities involved in teaching several grades simultaneously, along with the lack of materials and teaching preparation for the development of inter-disciplinary experiences that incorporate the knowledge and use of technology, considering the heterogeneity present in these contexts, increase the challenge [22]. In this context, we need to develop proposals that address the particularities of these territories and contribute to equal access to relevant educational experiences that incorporate the knowledge and use of technology [11]. Although proposals have been developed that seek to promote STEM learning in multigrade contexts, these have only been developed at a theoretical level, and it is necessary to explore the effects of their implementation in the classroom on learning development [6,22].

### 2.2. Robotics in STEM Education

Robots are technological tools of great interest at all educational levels, especially during the first years of schooling. Robotics presents an opportunity to introduce children from an early age to the world of technology and engineering, offering them significant opportunities to learn mathematical and scientific concepts, as well as to develop 21st century skills [13]. Educational robotics has the potential to help students explore the creative uses of technology, better understand the ever-evolving digital world, and reflect on its advantages and disadvantages [23]. It supports teaching approaches compatible with the demands of the 21st century, such as constructivism, and promotes the development of curricular learning in STEM areas [5,24].

Various studies have been undertaken that demonstrate the contribution of robotics to the motivation and participation of students, the development of their cognitive and social skills, computational and critical thinking, problem solving, creativity, collaboration, decision making, and mathematical and scientific skills [15,16,24,25]. However, the use of educational robotics is not yet fully incorporated into the compulsory school system. The use of robots to teach mathematics and science is not carried out systematically in all schools, being more an initiative of pioneer teachers [15,23]. Among the reported barriers to the widespread use of educational robotics as part of the regular curriculum are the lack of teacher preparation to make connections between robotics and STEM subjects and the failure to develop resources that are accessible in all educational contexts [5].

The reluctance of many teachers in taking on this new challenge for which they have not been prepared, accompanied by the lack of available teaching materials that respond to different educational contexts, increase the digital divide and allow educational robotics to be accessible only to a few privileged children, while the majority do not have similar opportunities [26]. This scenario highlights the need to develop proposals that promote the knowledge and use of educational robotics for the promotion of STEM learning in all educational contexts.

## 3. Course Design and Methodology

### 3.1. Background

Multigrade schools exist around the world as a way for countries to meet the education-for-all mandate when distances are too great to travel to an urban school every day or the population is too small. This teaching model, present in southeast Asia, Europe, North America, South America, and Australia, allows children to be educated who otherwise would not be able to go to school, helping them gain the knowledge and skills they need for a more promising future [7].

For example, in Chile there are 3299 rural educational establishments, of which 53.8% are in the most isolated areas of the country. Within the framework of the “Connectivity for Education 2030” program promoted by the Chilean Ministry of Education in 2021, more than 2000 of these educational establishments have benefited from high-speed internet access. These efforts seek to reduce the digital gap in rural schools, breaking down access barriers to technology as part of the national public policy. Although access to technology is a factor that influences the digital divide, it is not enough to reduce it. Because of significant technological developments in robotics and artificial intelligence, it is very likely that the digital divides will deepen, given that the skills necessary to implement these systems are more sophisticated and require advanced knowledge [27]. This poses the challenge of involving children from an early age in this type of educational experience, offering access to the latest generation of technological developments that will allow them to understand and participate in a world marked by rapid advances in these disciplines, improving their quality of life and that of their communities [5,28].

### 3.2. Methodology

A STEM methodology was used in this study. The STEM methodology is considered an active and interdisciplinary learning approach, based on problems or projects that require the application of knowledge from at least two STEM disciplines for their solution or development [19]. It promotes interdisciplinary learning of curricular topics in STEM areas, the development of 21st century skills, and the understanding of topics relevant to 21st century citizenship [29].

Considering the particularities of working in a multigrade classroom, this study used a STEM design based on the framework of STEM units in multigrade contexts [6,22] to promote the knowledge and use of educational robotics as a means for developing mathematics and science learning in several school grades at the same time. This framework, made up of two phases, is based on the development of great STEM ideas for the promotion of deep and balanced learning in the areas to be integrated, and differentiated instruction as a strategy to accommodate the heterogeneity of the classroom. Phase 1, in which the integration process takes place, involves the selection of a topic to be addressed, the selection of the big STEM idea, the identification of its main components through the learning horizon, and the evaluation of the educational decisions.

The second phase, in which the general planning of the unit is developed, includes the reorganization of the learning that will be part of the unit, the differentiation of the content to be addressed in each grade, the process that the students will undergo to achieve that learning, a description of what they will learn and do, and activity design.

#### 3.2.1. The Proposal to Incorporate Robotics for the Promotion of STEM Learning in Multigrade Contexts

**Figure 1:**
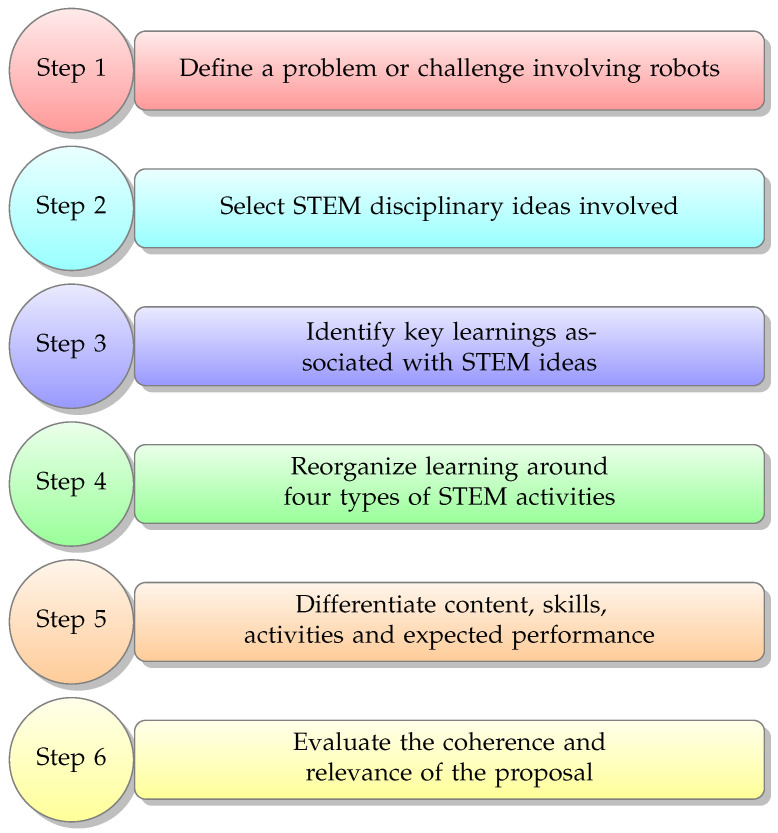
Proposal to incorporate the knowledge of robotics for the promotion of STEM learning in a multigrade context.

Based on the theoretical framework described above, we placed the knowledge and use of educational robotics for the promotion of STEM curricular learning in the context of a design problem that involved big STEM ideas. In this case, we selected one or more ideas within one or two STEM disciplines that pertained to our robotics problem or challenge. From this, we identified the key lessons associated with those ideas through the learning horizon of each area to be integrated. Next, we reorganized these lessons around four types of STEM core activities. We defined a preliminary activity to introduce students to the subject of study, motivate them, and activate previous knowledge for the development of the unit. We outlined the following activities: a core STEM activity to introduce students to the robotics challenge or problem and offer opportunities to develop a knowledge base that will allow them to situate their challenge or problem; exploration activities to offer students the opportunity to deepen their knowledge and develop their own skills in each of the disciplines that are part of the unit; and, finally, a consolidation and synthesis activity in which students respond to their robotics challenge or problem by applying the knowledge and skills developed during the unit. Once we organized the lessons in our unit around these four types of STEM activities, we established contents, skills, performances, and differentiated activities for each school grade, considering the curricular learning that must be developed. Finally, we evaluated the coherence and relevance of our proposal. Figure 1 illustrates the proposal to incorporate the knowledge and use of robotics for the promotion of STEM learning in a multigrade context.

Let us consider a robotics challenge as an example: creating and programming a robot to simulate a floodgate to control a river’s water flow. Once the problem is defined (step 1), it is necessary to identify and select an idea that covers STEM content—in this case, floods (step 2). Then, the critical learnings associated with the selected idea must be identified (step 3). These could include the scientific topics, such as the study of soil, climate, and ecosystems; mathematical topics, such as measurement and data analysis; or technological topics, such as robots and computational thinking. Then, it is necessary to organize the learnings into the four types of STEM activities (step 4), such as introducing floods and flood prevention and creating and programming a robot to simulate a floodgate. In step 5, the content, skills, activities, and expected performance must be differentiated for different school levels. For example, first grade students may learn about different weather characteristics, third grade students may learn about the relationship between weather and the seasons, and sixth grade students may learn about the effects of rainfall on the riverbed. Finally, in step 6, it is necessary to evaluate the coherence and relevance of the proposal for the classroom and make any necessary adjustments.

#### 3.2.2. Participant Criteria for the Composition of Working Groups

All the students in grades 4, 5, and 6 of a multigrade rural school located in an austral area of southern Chile participated in this study. The sample consisted of 12 students, representing around 50% of all students at the school. Specifically, three fourth-graders, three fifth-graders, and six sixth-graders. The age range of the students was between 9 and 13 years, including eight females and four males. None of the students had previous experience with any educational robotic kits, and only two of them knew what a robot was and recognized their presence in our daily lives, but did not know how they worked. All the tutors signed an informed consent declaration describing the objectives of the study and the activities to be carried out and authorizing the participation of their children. The students were also informed and signed an informed assent declaration.

The work in the classroom was based on group work by school grade and the educational needs of the students. This differentiation strategy made it possible to work with several groups at the same time, promoting collaborative learning among peers, communication, the development of curricular learning specific to the level, and attention to the needs of each student [6,22]. Thus, three work teams were formed. The first of them contained three fourth-grade students; the second contained five sixth-grade students; and the third contained three fifth-grade students, and one sixth-grade student who had special educational needs and required curricular adjustments.

#### 3.2.3. Development of the Sessions

Using the theoretical framework described above, we designed the STEM unit.

- Step 1. We focused the development of our unit on an achievable challenge for all school grades that would participate in the unit: the challenge of designing and assembling a mobile robot that would allow objects to be dragged using the Lego WeDo 2.0 kit.
- Step 2. Three great STEM ideas that were understandable by all students were defined. In science, we considered force and its effects; in mathematics, the measurement of magnitudes; and in technology, what is a robot and programming in a robotics context.
- Step 3. From this, a review of the primary education study plans in these areas was undertaken and the key curricular lessons associated with the challenge were identified that could be addressed in the school grades involved. In science, these comprised the concepts of force, mass, and friction, and in mathematics, the measurement of length, mass, and volume. Since there was no curriculum for robotics in primary education, studies aimed at promoting the knowledge and use of educational robotics in primary education were used as a basis [15,16].
- Step 4. This learning was reorganized around four types of STEM activities, and the knowledge, skills, activities, and expected performance for each school grade were established. We defined (1) a preliminary STEM activity to investigate what a robot is and its presence in our daily lives, (2) a central STEM activity to design a mobile robot that drags an object, (3) STEM exploration activities to assemble and program a robot that can drag objects over a certain distance, and (4) a consolidation and synthesis activity to improve their prototype.
- Step 5. Based on the curricular learning established for each school grade, activities and expected performances were established. For example, fourth grade students were asked to search for information about what a robot is, its uses, and the characteristics of a mobile-type robot using a technological object, using keywords given by the teacher. The fifth and sixth graders did this by checking different websites autonomously.
- Step 6. Finally, the coherence and relevance of the proposal as a whole were evaluated, and the corresponding adjustments were made.

Table 1 describes the planning of the sessions developed during the unit.

**Figure 2:**
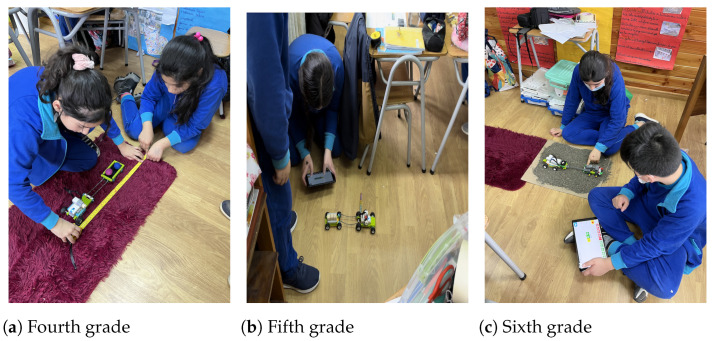
Fourth session exploration activities.

As noted in Table 1, for each of the sessions, guiding questions were established to address content, skills, activities, and differentiated performances for each school grade. For example, in Session 4 (See Figure 2), fourth-graders determined the maximum load their robot could pull by carrying balls and cubes of playdough with the same mass. The students determined the path traveled by their robot with different loads, on the same surface and on a surface with different levels of roughness. Here we focused on understanding the scientific concept of mass and its properties and the idea of friction, and on the mathematical measurement of length and mass. During this session the fifth-grade students focused on the study of friction and the operation of their robot through the measurement of length and mass. To do this, the students compared the effects of loading their robot with wooden and stone cubes with the same edge on two surfaces with different roughness. Here the question focused on what aspects affected the movement of their robot. During this session the sixth-grade students focused on determining the number of cubes that their robot could drag along the floor. To do this, they experimented by dragging cubes of wood and stone with the same edge on three surfaces with different levels of roughness. This activity allowed the students to reflect on the capabilities of their robot based on its structure and components, deepening prior knowledge associated with measuring mass and length and making sense of measurement.

The unit was developed in six sessions of 120 min carried out twice each week. These were implemented by one of the researchers in the classroom in the presence of the teacher responsible for the group and recorded on video.

Table 2 lists the objectives of each session and the resources and materials used in them.

#### 3.2.4. Methods and Tools of Analysis

Two research questions were posed in this study:

- In what way does the knowledge of robotics promote the curricular learning of mathematics and science in rural multigrade classrooms at the same time?
- What are the perceptions of students who do not normally have access to these types of experiences about employing the knowledge and use of robotics to learn mathematics and science?

To answer these questions, the authors used an exploratory qualitative methodology. Two data collection techniques were used: the focus group and the semi-structured interview. To analyze how the knowledge and use of educational robotics promoted the curricular learning of science and mathematics in several school grades at the same time, two student focus groups were implemented during the development of the unit. The sessions were videotaped, transcribed, and analyzed through inductive thematic analysis [30]. Descriptive codes were established, reviewed by the researchers together, and organized into themes based on their similarities by type and frequency.

**Figure 3:**
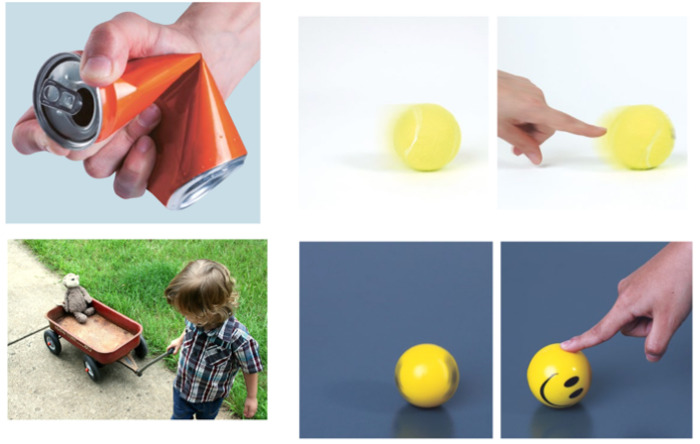
Some images presented to the students to explore their knowledge.

The first focus group was implemented at the beginning of Session 5, after the students delved into the study of force and its effects. Here the focus was the understanding of developed disciplinary knowledge. Students were presented with different images of objects (see Figure 3) that require the application of a force to move or stop, and open questions were asked of the group, such as: What should be applied to deform the beverage can and the elastic? Or to move the carts? What should we do to make the ball move slower? What are some of the effects of force?

Later, the students were presented with images of the experiments they conducted making their robot drag loads in different conditions. Here the focus was on exploring the ideas developed around friction and mass. The students were asked: On which surface does the robot travel a greater distance? Does the robot move a greater distance when carrying a load of great mass or one of low mass? What does the distance my robot travels depend on each time?

The second focus group was implemented at the end of the unit. Here the perceptions of appropriation of knowledge on the topics addressed in the unit around robotics, programming, science, and mathematics were explored. Students were asked five key questions: What did they learn during the development of this unit? What mathematics did we use during these activities? What did we learn in science? What did we learn about robotics? What did we learn about programming?

To explore the students’ perceptions of the use of knowledge and the use of robotics as a way of learning mathematics and science, semi-structured interviews were conducted. The interviews were held at the end of the unit; they were audio recorded, transcribed, and an inductive thematic analysis was applied [30]. As in the previous case, descriptive codes were established that were reviewed jointly by the researchers and organized into themes based on their similarities by type and frequency. Here, five key questions were included: Did you like learning about robotics? What did you think of using robots to learn math and science? Do you think that working with robots helped you learn about math and science? Would you like robots to be used regularly in math and science classes? Do you think it is important that all children learn about robotics?

## 4. Results

This study explored how the knowledge of robotics contributed to promoting the curricular learning of science and mathematics in various school grades and the students’ perceptions of the use of robotics to learn mathematics and science.

### 4.1. Robotics as an Opportunity to Promote STEM Learning in Multigrade Contexts

To analyze how the knowledge of robotics promoted the curricular learning of science and mathematics in several school grades at the same time in answer to research question 1, we analyzed the observations of the students in the focus groups. The interview protocol used in the first focus group focused on exploring the understanding of disciplinary knowledge developed by the students, whereas the second focus group was aimed at exploring student perceptions of the appropriation of knowledge on the topics addressed in the unit around robotics, programming, science, and mathematics.

The answers that the students gave to the questions posed in the first focus group were organized into three categories, as shown in Table 3.

As seen in Table 3, most of the students (70%) understood the effects of forces, both in the deformation that they can cause in objects, and in the changes in their state of movement (whether they are placed in motion or change their speed while in motion). This was reflected in the students’ answers when asked, for example, what should be applied to deform the can and the elastic or to make a moving ball roll faster, slow down, or stop. Typical responses included: “force”, “a force”, “push it”, “a force in favor”, “a force against”, “to the left”.

Regarding the friction that occurs on surfaces in relative motion, we observed that students (100%) understood that when an object moves on a surface, it presents a resistance to movement. This was reflected in the answers they gave when asked what happened to the speed of the car when it moved from the floor to the carpet, what should be applied to make a body move slower, whether there was more or less friction on the floor. Here the answers were of the type: “slower”, “a force against”, “friction”, “less”.

Regarding mass and its interpretation as a resistance to change in the movement of bodies, we observed that 50% of the students associated mass with a measure of resistance to movement. This was evidenced when they were asked, for example, in which case would a car roll more under the same applied impulse: a car loaded with flowers or the same car loaded with a child and her backpack. Here, the answers were “the cart with the flowers”, “the cart with the flowers, because it has less mass”.

The answers that the students gave to the questions posed in the second focus group were organized into four main categories of perceptions of the appropriation of knowledge around disciplinary themes developed during the unit (see Table 4).

As seen in Table 4, the students expressed perceptions of appropriation of knowledge regarding all the disciplinary topics developed during the unit. The topic that was most frequently reported was the measurement of magnitudes in which perceptions of appropriation of knowledge associated with the measurement of mass, volume, and length were reported, as well as the use of specific units. Students indicated they had learned “how to measure the volume”, “the cube unit”, “length”, “mass and volume”. Second, the theme of strength and its effects emerged. Here learning perceptions associated with force and its effects were reported, for example, the students learned about “friction”, “force”, “effects”. The third category included self-learning perceptions associated with programming. Responses included that programming allows “giving orders to the robot”, determining “the time that the robot can move and make it go back”, “that it can go faster”, “put colors or music”. The fourth emerging category was robotics. Here learning associated with the assembly, operation, and main components was reported, such as: “How to assemble robots”, “What was the controller, the sensors”, “About motors, sensors and controllers” and “How to control robots”.

### 4.2. Perceptions about the Use of Robotics to Learn Mathematics and Science

To explore the students’ perceptions of utilizing knowledge of robotics as a way of learning mathematics and science and to answer research question 2, the responses of the 10 students interviewed were qualitatively analyzed. Between three and four response categories were established depending on the question. Thus, for example, the answers to the question: Did you like learning about robotics? were organized into three categories in which a positive assessment of the experience was made. In the first category were the responses of the students who had positive assessment of learning robotics because of the opportunity to learn new things (5 out of 10 students): “Yes, I loved it, because I learned new things about Legos, about motors, sensors, all that” or “Because there we can learn about robots, what their parts are”. The second category contained responses that were positive because it was entertaining and they enjoyed doing the unit (4 out of 10): “Yes, a lot, because I liked the robots, and this one..., they are charming, they are entertaining”, “Yes, it was really fun”. Finally, the third category included both the taste for robotics and learning new things: “Because it is entertaining and something new” (1 student).

Regarding the students’ perceptions of using robotics to learn mathematics and science, we observed that the majority (8 out of 10 students) had a positive perception because they found it more entertaining and enjoyable. This was evidenced when students pointed out, for example: “Good, because I don’t like math”, “Entertaining, it’s better than with numbers”, “Funny”, “I thought it was very nice because we learned about length and stuff, what can get in, which can load the robot and stuff.” One student admitted that she did not feel that it helped her much, and another did not answer the question. Positive perceptions of using robotics to learn mathematics and science coincided with the students’ perceptions of the learning achieved during the unit. When asked if they considered that working with robots helped them learn about mathematics and science, we observed that the majority (8 out of 10) indicated that it did help them explicitly (“Yes, it helped a lot”) or indicated things they learned (“The force made the robot go forward”). One student indicated that it helped him a little, and another did not feel that it helped him. However, when asked if they would like robots to be used regularly in mathematics and science classes, all students said yes, arguing that this way was more fun and reduced the difficulty of mathematics: “Yes, because math is hard”, “Yes, because it is very entertaining”, or “Because those subjects are boring”.

All the students interviewed considered it important that all children learn about robotics because it is useful training for them and offers learning opportunities, pointing out, for example: “Yes, because that way we all have how to learn something”, ”Yes, so that they understand things better”, “Yes, a lot, because, um…, they learn things like length, weight and stuff”.

## 5. Discussion

Based on the data obtained and considering the two research questions, we now discuss the contribution of the knowledge and use of educational robotics for the promotion of the curricular learning of science and mathematics in various school grades.

The first question posed in this study explored how the knowledge of robotics promoted curricular learning of mathematics and science in rural classrooms containing several school grades at the same time. The results obtained in this study suggested that use of the knowledge of robotics to promote the curricular learning of mathematics and science in several school grades simultaneously is a viable response to the challenge of adapting the curriculum for teaching several grades at the same time.

Understanding of the science concepts involved was frequently evident in the students’ responses, but for mathematics it was not as evident. All the students who participated in the unit managed to understand the concepts derived from science, recognizing them, and applying them in different situations in which they were involved. The promotion of involved mathematical learning was evidenced more clearly in the fourth and fifth grades, but not in the sixth grade. All students recognized the importance of measuring the magnitudes involved in the unit to solve the challenge, as well as how to measure them and the associated unit of measurement. The fourth and fifth grade students satisfactorily understood the mathematical concepts because they were more evident to them. These concepts had more meaning for the students when they visualized them more concretely. In contrast, the sixth grade students found it more challenging to visualize the concept of volume calculation. However, because of the limited time available for the development of the unit, it was not possible to go deeper to determine to what extent these concepts were understood at the different school levels.

Other studies have reported inconclusive results on the role of robotics in the development of mathematical learning; thus, it is necessary to conduct more research in the area to evaluate the effects of robotics on mathematical learning gains [31]. These results represent an initial exploration of the impact of robotics on STEM learning in multigrade contexts. These findings align with other studies conducted in traditional school settings, showing that educational robotics can improve students’ understanding of scientific concepts [16,24,30,32]. It is worth noting that multigrade schools, which are often located in isolated areas and may have as few as three or four students, pose a unique challenge for research in this field. In order to make more meaningful comparisons between groups, it is necessary to increase the sample size, considering the geographic and social reality of each multigrade school involved in the study.

It is important to note that there are other robotics kits, such as Lego Mindstorms or Coding Root Robots, that can be used to support learning in science and mathematics. However, not all these kits are suitable for use in a multigrade classroom without guidance. Each robotics kit is designed to be used by a specific age range of users and the programming languages and concepts involved can vary significantly between kits. For example, Lego Wedo 2.0 is for ages seven and up, Lego Mindstorms is for ages ten and up, and Coding Root Robot is for children ages three and up. When choosing a robotics kit for a multigrade classroom, it is necessary to consider the age range and heterogeneity of the students and to select a kit that is appropriate for their level and that offers the option to adjust the difficulty as needed.

This study also suggests new research avenues for exploring robotic simulator use in multigrade contexts for STEM education. Various simulators could be used for this purpose [33], such as the Tactode Simulator for working on challenges related to constructing polygons [34] or Robo Sim for locating points on a plane [5]. However, the appropriateness of using these resources will depend on the specific goals of the STEM unit being developed. Some activities, such as measuring mass or determining the maximum load a mobile vehicle can carry under different conditions, may not be well-suited to a simulated environment due to their practical, experiential nature.

The second question explored the students’ perceptions of utilizing the knowledge and use of educational robotics to learn mathematics and science. The results suggested positive perceptions of the use of robotics for learning mathematics and science. All students noted that they would like robots to be used regularly in mathematics and science classes. These findings are consistent with similar studies indicating the potential of educational robotics in motivating students to learn mathematics and science [15,31]. They also demonstrate the need to progress the widespread and regular use of educational robotics in the classroom. One factor that hinders this process is teacher preparation. More research is required to determine the best way to prepare teachers for this task and support them in establishing connections between the subject and robotics, as well as to develop resources that are accessible in all educational contexts [5,26].

## 6. Conclusions

This proposal provides a way to reduce the inequalities that rural schools face in the knowledge and use of the latest technologies in the classroom and the development of authentic and relevant learning experiences for the citizenship of the 21st century. In a society that aims to reduce inequality, it is essential that all children, regardless of their context, be exposed to the wonders of educational robotics [26]. This study offers an alternative to bring educational robotics closer to the rural school, incorporating its knowledge with a STEM approach to promote curricular learning in mathematics and science in several school grades at the same time.

## Figures and Tables

**Table 1:** Session plan. S indicates the session number.

S		Activity	Guide Question	Developed
1	(120 min)	STEM Preliminary	What is a robot and what is it for? What are the main characteristics of a mobile robot?	Team formation. Research on what a robot is, what they are for and the main characteristics of a mobile type of robot. Plenary
2	(120 min)	STEM central	What is required for objects to move? What components does a mobile type of robot require to make it move?	Video analysis of objects being pushed and pulled, explaining why they move. Comparison of the effect of the application of a force on objects of the same size and shape and different mass, objects of the same size and mass but with wheels and without them. Description of the effect generated by a motor from the Lego WeDo kit on a motionless object, assembling and programming a rotating propeller. Design and presentation of a robot prototype that can drag an object, including key ideas.
3	(120 min)	STEM exploration 1	How do objects move? How to instruct a robot to move?	Build a sturdy robotic vehicle that can be programmed to pull an object, using the LEGO WeDo kit. Exploring the effects of the order of instructions in a program. Creating a program for the robot to move a given distance.
4–5	(240 min)	STEM exploration 2 and 3	How to determine the distance my robot can drag an object? What does the load limit that my robot can drag depend on?	Determining the maximum load your robot can pull: -measuring the mass of various objects that your robot can drag on surfaces with different roughness, depending on the grade level. -measuring/calculating the volume of your load with non-standardized and standardized units, according to the school grade, on surfaces with different roughness. -determining the distance covered in each case in centimeters.
6	(120 min)	Consolidation and synthesis	What aspects should I consider to improve the towing capacity of my robot?	The teams: Analyze limitations of their robots. Redesign their prototype. Present their redesign justifying the changes made to improve it.

**Table 2:** Objectives of each session and resources used. S indicates the session number.

S	Objective	Resources and Materials
1	**Understand that:** Robots are programmable machines to perform autonomous tasks and are used for different activities. Robots have main components that allow them to perform tasks autonomously and interact with the environment.	Tablet, projector and computer
2	**Understand that:** The movement of an object at rest is a consequence of applying a force on it. When applying the same force on an object at rest with different masses, its movement will be different. Robots have significant components such as controllers, motors, and actuators that allow them to move. Robots are programmed with instructions to accomplish a particular task autonomously.	Lego bricks, stones, Lego Wedo 2.0, projector, tablet, computer and work log.
3	**Understand that:** A program is a specific sequence of instructions or steps that allow us to perform a specific task. Robots are programmed with instructions that the robot executes in order. Each instruction has a specific meaning, and the order of the instructions affects the overall actions of the robot. **Be able to:** Build in teams a robust robotic vehicle using the LEGO Wedo kit, which can be programmed to drag an object. Analyze the consequences of the action of a program modifying the order of the icons, power, time, and direction of rotation. Create and execute a program so the robot can move a certain distance.	Lego Wedo 2.0, projector, tablet and work log.
4	**Understand that:** The force of friction depends on the type of surfaces in contact. Movements can take various trajectories. Objects have properties that can be measured, such as length, mass, and capacity. Mass is the physical magnitude that indicates the amount of matter a body contains, regardless of where the body is located. Weight is the force exerted by gravity on a mass. Mass is usually measured using a scale in standardized units. Some objects maintain their mass despite transforming. **Be able to:** Measure length, mass, and capacity using conventional or unconventional measurements. Use appropriate measuring instruments.	Scale, ruler, measuring tape, Lego Wedo 2.0, projector, tablet, clay, wood cubes, stone cubes, sand, carpet and work log.
5	**Understand that:** Volume is the amount of space occupied by bodies, and its unit of measurement is the cubic meter ($m^{3}$). In the same type of body, the smaller the volume, the smaller the mass, and the larger the volume, the larger the mass. The relationship between mass and volume is different when the type of body changes.	Scale, ruler, measuring tape, Lego Wedo 2.0, projector, tablet, clay, wood cubes, stone cubes, sand, carpet and work log.
6	Redesign prototype considering key aspects that allow improving its performance.	Lego Wedo 2.0, tablets and work log.

**Table 3:** Categories of analysis focal group 1.

Categories	Description	Frequency	Expected Frequency
Effects of forces	The application of a force generates deformations in bodies and changes in their speed	21	30
Surfaces and opposition to movement	Surfaces resist the movement of objects, depending upon the type of surface	10	10
Mass and resistance to change in motion	The mass can be interpreted as a resistance to the change of movement	5	10

**Table 4:** Categories of analysis focal group 2.

Categories	Description	Frequency	Expected Frequency
Force and its effects	Includes perceptions of appropriation of knowledge about the concept of force and its effects	8	10
Measurement of magnitudes	Includes perceptions of appropriation of knowledge about the measurement of length, mass, and volume, as well as of specific measurement units	17	20
Programming	Includes perceptions of appropriation of knowledge about what a program is and its effects and how to program the robot.	7	10
Robotics	Includes perceptions of appropriation of knowledge about the assembly of robots and their main components.	6	10

## Abbreviations

The following abbreviations are used in this manuscript:

STEM	Science, Technology, Engineering and Mathematics
